# Clinical Utility of a Feedback Device in High-Quality Cardiopulmonary Resuscitation: The Guardian Angel

**DOI:** 10.3390/jcm15082839

**Published:** 2026-04-09

**Authors:** Jaime Fernández-Sarmiento, Andrés Rolando Sanmiguel-Benavides, Juan Pablo Contreras, Alirio Bastidas, Juan Carlos Trujillo, Juanita Uribe, Daniel Botero-Rosas, Eduardo Tuta-Quintero

**Affiliations:** 1 Department of Critical Care Medicine and Pediatrics, Fundación Cardioinfantil—Instituto de Cardiología, Bogotá 110131, Colombia; 2 School of Medicine, Universidad de La Sabana, Campus del Puente del Común, Km. 7, Autopista Norte de Bogotá, Chía 250001, Colombiajuanconhe@unisabana.edu.co (J.P.C.); eduardotuqu@unisabana.edu.co (E.T.-Q.); 3 Department of Internal Medicine and Epidemiology, Universidad de La Sabana, Campus del Puente del Común, Km. 7, Autopista Norte de Bogotá, Chía 250001, Colombia

**Keywords:** cardiopulmonary resuscitation, chest compression quality, feedback device, infrared-based device, simulation study, healthcare personnel

## Abstract

**Background/Objectives:** High-quality cardiopulmonary resuscitation (CPR) is essential for improving clinical outcomes in patients experiencing cardiac arrest. Feedback devices designed to guide CPR must offer real-time feedback on chest compression quality for use by both medical personnel and laypersons. To describe the impact of using an infrared-based device to provide feedback on chest compression quality during CPR performed by healthcare personnel. **Methods:** A before-and-after study conducted in a simulated hospital from February 2019 to February 2020 evaluated the difference in the percentage of adequate chest compressions in depth and rate. This was assessed by comparing the number of adequate compressions to the total performed in one minute, both with and without the use of a feedback device. **Results:** A total of 90 participants were recruited, with an average age of 31.2 years (SD: 8.86), of which 60% (54/90) were women. In infants and schoolchildren/adults, high-quality compressions improved by 66% (85% vs. 19%; *p* < 0.001) and 54% (79% vs. 25%; *p* < 0.001), respectively, when guided by the GA compared to the control group. In participants without training and experience in CPR, the use of the Guardian Angel achieved high-quality compressions of 45% (10/22) and 66% (25/42), respectively, when evaluating depth for the schoolchildren/adults group. The use of the device during CPR, even in situations of moderate or severe fatigue, resulted in an improvement in high-quality compressions in terms of frequency, reaching 73% (16/22) in infants and 68% (15/22) in schoolchildren/adults. **Conclusions:** The Guardian Angel improves both the frequency and depth of compressions in participants without previous CPR experience or training. Additionally, the device demonstrated notable improvements in compression frequency, even in situations of moderate or severe fatigue.

## 1. Introduction

High-quality cardiopulmonary resuscitation (CPR) is essential for improving clinical outcomes in patients experiencing cardiac arrest [1,2]. The quality of chest compression components, such as hand position, rescuer and victim positioning, and compression rate and depth, are crucial for enhancing clinical outcomes like survival and neurological recovery [1,3,4]. International guidelines recommend the implementation of feedback devices during CPR to enhance the consistency and quality of chest compressions, potentially impacting morbidity and mortality positively, irrespective of the operator or setting [4,5].

Feedback devices designed to guide CPR must offer real-time feedback on chest compression quality for use by both medical personnel and laypersons [6,7,8,9]. Various devices have been described for evaluating chest compression quality in clinical and medical training settings, some of which can interface with devices like smartwatches, defibrillators, and memory cards, for both in-hospital and out-of-hospital use [8,10]. Vahedian-Azimi et al. [6] demonstrated that the feedback device Cardio First Angel^TM^ (CFA; INOTECH, Nubberg, Germany) improved CPR quality and consistency in participants guided by the device compared to the control group (*p* < 0.001).

There is limited available medical literature on feedback devices utilizing infrared technology to enhance CPR quality in clinical and medical training settings [1,2,9,10]. The objective of this research is to evaluate the impact of using an infrared-based device to provide feedback on chest compression quality during CPR performed by healthcare personnel.

## 2. Methods

An analytical study of the before-and-after type was conducted between February 2019 and February 2020 at the simulated hospital “Camilo Cabrera” of the Fundación Cardio Infantil—Instituto de Cardiología of Bogotá, Colombia. This simulation center is dedicated to conducting training in basic and advanced CPR, as well as other simulation courses. The manikins used are approved by the International Liaison Committee on Resuscitation (ILCOR) and the American Heart Association (AHA) [1,2].

### 2.1. Outcomes

The primary outcome of this study was the objective quality of chest compressions, defined according to AHA guidelines as a compression depth of 5–6 cm and a rate of 100–120 compressions per minute [1,2]. Quality was expressed as the percentage of compressions meeting these criteria during each one-minute CPR cycle.

A secondary outcome was the participant’s perception of performing high-quality CPR. This variable was assessed immediately after each CPR cycle through self-report and reflected the rescuer’s subjective evaluation of their performance, independent of the objective measurements recorded by the device. This measure was included to explore the potential influence of real-time feedback on self-confidence and self-awareness during resuscitation maneuvers.

### 2.2. Eligibility Criteria

The study population consisted of individuals over 18 years old willing to perform CPR under the supervision of the feedback device. Subjects with any type of auditory, visual, cognitive, or physical disability hindering the performance of chest compressions were excluded. This research was approved by the research committees of the Universidad de la Sabana and La Cardio. All participants signed informed consent authorizing their inclusion in this study.

### 2.3. Variables

Variables studied included educational level; history of CPR training; physical fatigue before, during, and at the end of the test; and healthcare professionals in training and/or graduates. Compression quality was analyzed according to AHA guidelines [1,2]: depth between 5–6 cm and a rate of 100 to 120 compressions per minute.

### 2.4. Device Design and Laboratory Testing

**Figure 1:**
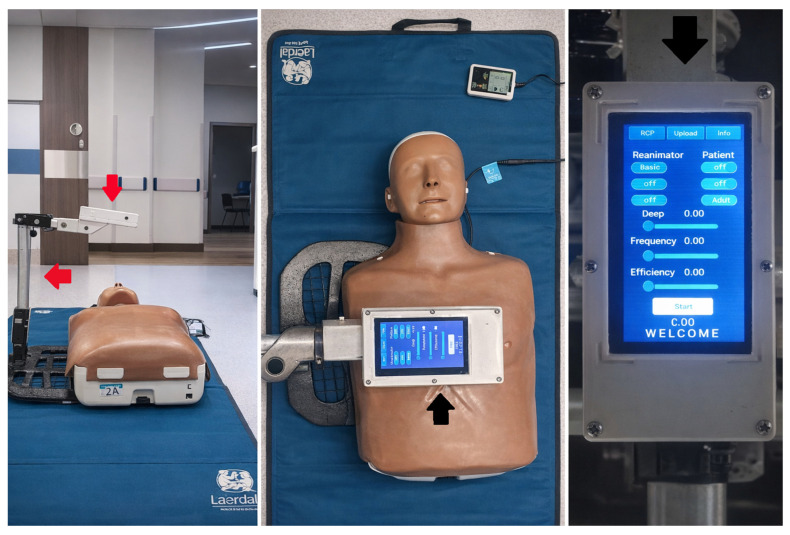
Real-time infrared feedback device Guardian Angel. Notes: Version 1.0 is depicted. Red arrow: shows the Guardian Angel on a practice mannequin. Black arrow: indicates the LED screen displaying variables such as Depth, Frequency, and Efficiency.

The feedback device was named “Guardian Angel,” which utilizes infrared technology to measure the depth and frequency of effective chest compressions according to the latest ILCOR and AHA recommendations. Feedback is provided in real-time through direct visualization on a 2.4-inch Thin Film Transistor Liquid Crystal Display Arduino^TM^ screen, allowing the rescuer to adjust maneuvers to achieve goals (Figure 1). A Sharp^®^ proximity sensor (GP2Y0A21YK) based on an emission-reception system in the infrared spectrum (lower than radio waves and higher than light) adapted to a microcontroller was used in its development. This technique provided high-precision distance measurement within a range of 10 to 80 cm. The general characteristics of the Sharp^®^ GP2Y0A21YK (Sharp Corporation, Osaka, Japan) are described in Figure S1. The open-source development board (Arduino^TM^ MEGA 2560; Monza, Italy) with an Atmega2560 microprocessor featuring input-output (I/O) pins was adapted to the infrared measurement sensor to determine object distances. It also has a 16 MHz crystal oscillator and 256 k flash memory.

Measurement results are reflected on a color touchscreen Liquid Crystal Display, displaying information about compression frequency and resuscitation time. This same information is transmitted via Bluetooth technology to a Smartphone through an Android application developed using Microsoft Visual Studio 2015. This application also facilitated device calibration and reset of the timer or adjustment of measurement ranges according to age group.

### 2.5. Study Procedure

Equipment calibration was performed with a pilot test to ascertain the correct frequency and depth of compressions, conducted with each rescuer. Healthcare professionals were given a demonstration of how the device operates during a CPR cycle. Included volunteers performed compressions on manikins initially without viewing the device. Subsequently, they were asked to rest for double the time they spent performing chest compressions. Once the rescuer reported feeling physically able to continue, a new cycle of compressions was performed while observing the device, allowing for correction of compression frequency and depth. Before starting sample collection, it was ensured that the manikin was on a rigid surface, hand positioning was correct, and the infrared device was positioned on the support with the infrared light on the back of the rescuer’s hand.

### 2.6. Statistical Analysis

Data were collected using the Research Electronic Data Capture (REDCap) electronic data capture software [11] and analyzed using SPSS version 25 (IBM, Armonk, NY, USA). Descriptive analysis was performed for quantitative variables using measures of central tendency and dispersion according to their distribution, while absolute and relative frequencies were used for qualitative variables [12]. The Chi-square test was used for the comparison of categorical variables and Fisher’s exact test was used in cases of fewer than 5 participants per cell [12]. The percentage of compressions adequate in depth was calculated with the number of compressions adequate in depth over the total number of chest compressions performed in one minute. The percentage of compressions adequate in frequency was calculated with the number of compressions at an adequate rate over the total number of compressions. A significance level of *p* < 0.05 was considered [12].

### 2.7. Ethical Considerations

The studies involving human participants were reviewed and approved by the Ethics Committee of the Fundación Cardio Infantil—Instituto de Cardiología and Universidad de La Sabana (68—18 May 2018). Although it is considered minimal risk research, in which no intervention will be carried out, informed consent was collected.

## 3. Results

A total of 90 participants were recruited, with an average age of 31.2 years (SD: 8.86), of which 60% (54/90) were women. A total of 76% (68/90) of participants had previous training in high-quality CPR, while 58% (52/90) had clinical experience in resuscitation. The general characteristics of the population are described in Table 1.

In total, 96.7% of the participants did not report previous fatigue before starting CPR; 65.6% (56/90) and 63.3% (57/90) of the participants reported moderate fatigue during and after CPR. The perception of performing high-quality compressions was 97% (87/90) in the group that used the device, compared to 63% (57/90) in the control group (Table 2). In infants and schoolchildren/adults, high-quality compressions improved by 66% (85% vs. 19%; *p* < 0.001) and 54% (79% vs. 25%; *p* < 0.001), respectively, when guided by the GA compared to the control group.

In participants without training and experience in CPR, the use of the Guardian Angel achieved high-quality compressions of 45% (10/22) and 66% (25/42), respectively, when evaluating depth for the schoolchildren/adults group (Table 3). The use of the device during CPR, even in situations of moderate or severe fatigue, resulted in an improvement in high-quality compressions in terms of frequency, reaching 73% (16/22) in infants and 68% (15/22) in schoolchildren/adults.

## 4. Discussion

This study evaluated the use of feedback devices during CPR. The Guardian Angel device improved the quality of compressions in terms of frequency and depth in infants and schoolchildren/adults. Even without previous CPR experience, the device increased high-quality compressions in terms of depth. Additionally, its use demonstrated significant improvements in compression frequency, even in situations of moderate or severe fatigue. It is important to note that this perception reflects a subjective self-assessment, rather than the performance recorded by the device. The inclusion of this variable was intended to explore whether real-time feedback can improve rescuer confidence and performance awareness [4,5,10]. This may have practical implications, as perceived confidence and competence can influence adherence to resuscitation guidelines in real-world clinical settings [4,13,14]. However, discrepancies between perceived and objectively measured performance should be interpreted with caution, as subjective assessment does not necessarily correlate with the actual quality of CPR.

Identifying and estimating compression frequency and depth during high-quality resuscitation requires the use of a feedback device [13,14]. However, the quality of chest compressions is rarely objectively measured. Chelladurai et al. [15] evaluated an audiovisual CPR feedback device to improve the proportion of chest compressions meeting AHA guidelines. The use of the feedback device improved depth quality by 46.4% (85% vs. 38.6%) (*p* = 0.003) and frequency by 47.1% (86.4% vs. 39.3%) (*p* = 0.001) compared to the control group. Like our study, without audiovisual feedback, a low proportion of participants met international guidelines for compression depth and frequency.

Wagner et al. [16] evaluated the quality of resuscitation and ventilation performed by healthcare personnel in the neonatal population using randomized feedback devices. It was found that chest compression and ventilation quality were better when a feedback device was used. However, participant attention was emphasized on the manikin and other equipment rather than the feedback device, and subjective workload increased. Although in our study, participant attention loss due to constant device assessment was not a described variable, we believe that due to the structural, foundational, and location characteristics of the Guardian Angel, this was not a confounding variable in our results.

The quality of CPR technique is crucial and should be based on variables such as compression depth and frequency [17,18]. Additionally, the use of appropriate compression force and depth is important to minimize associated injuries during resuscitation. Factors such as physical fatigue, educational level, previous training, experience in the technique, and duration of the cycle per rescuer may influence compression quality [19]. Our results highlight the utility of Guardian Angel use in participants without previous CPR training or experience, even in situations of moderate or severe fatigue.

The advantages of the Guardian Angel are particularly highlighted by the accuracy of its infrared sensors compared to ultrasound sensors, especially at medium and long distances [20,21]. Infrared sensor calibration is performed using a look-up table, ensuring precise measurement of the detected distance. Feedback is provided in real-time through a direct display screen. In addition to its ease of use, low cost, and accessibility, the device offers reliably focused visual attention during resuscitation. This leads to a notable improvement in compression quality, which could result in a more favourable prognosis for patients [20,21].

The use of real-time feedback devices to improve CPR quality increases the likelihood of achieving recommended compression rate and depth targets. Commercially available systems, such as the Laerdal QCPR platform and ZOLL Real CPR Help [22,23,24], typically rely on accelerometers, force sensors, or impedance-based measurements integrated into defibrillators or manikins. While these devices have demonstrated improvements in compression metrics, they often depend on proprietary hardware, may require specific adhesive patches or electrodes, and are often limited to institutional settings due to cost and infrastructure requirements [22,23,24]. In contrast, the Guardian Angel uses an infrared proximity sensor (Sharp^®^ GP2Y0A21YK) coupled to an open-source Arduino^TM^ platform to measure compression rate and depth without requiring integration into a defibrillator system. Its real-time visual feedback via an LCD screen and Bluetooth connection to a smartphone app allows for immediate technique correction in all age groups, including babies, as demonstrated in our before-and-after simulation study.

### Limitations

One of the main strengths of this study is its prospective before-and-after design, which allowed for the evaluation of the impact of real-time feedback on CPR quality in a relatively large sample encompassing different age groups. Nevertheless, the findings should be interpreted with caution, as this was a single-center study and the results may not be generalizable to broader clinical settings at the national or international level.

An additional limitation is that the sample was predominantly composed of healthcare professionals with prior CPR training and experience. Although participants received a standardized demonstration of the device’s operation and underwent individual calibration before data collection, their familiarity with resuscitation principles and simulation-based environments may have facilitated adaptation to the real-time feedback system [4,5]. As a result, the observed improvements could overestimate the performance achievable among individuals without healthcare training.

The Guardian Angel is primarily designed for controlled clinical and professional prehospital environments, such as emergency departments, intensive care units, and advanced life support ambulances [1,2,25]. However, we acknowledge that its current size represents a limitation, particularly in scenarios requiring high portability or rapid deployment by lay responders. In the settings, the device may complement manual or mechanical CPR by providing real-time monitoring and feedback. While increasingly compact feedback devices may be more practical for non-professional rescuers and large-scale community distribution, Guardian Angel is positioned as a professional-grade monitoring solution, prioritizing precision and integration within structured resuscitation systems over maximum portability [1,2,25].

An additional limitation is that implementation-related variables, such as device deployment time, ease of routine use, and feasibility in real-world acute resuscitation situations, were not measured prospectively. Therefore, no conclusions can be drawn regarding operational challenges or integration in real-world settings. For future research, it is necessary to evaluate rates of spontaneous circulation recovery in patients with cardiac arrest, both in hospital and out-of-hospital settings, using the Guardian Angel [1,2,25]. These studies should continue to accumulate evidence on the use of feedback devices to achieve high-quality CPR, as established by worldwide guidelines, and should be applicable regardless of the rescuer’s profession [1,2,22,23,24,25].

## 5. Conclusions

The Guardian Angel improves both the frequency and depth of compressions in participants without previous CPR experience or training. Additionally, the device demonstrated notable improvements in compression frequency, even in situations of moderate or severe fatigue. This highlights the importance of using feedback devices during cardiac arrest to enhance CPR quality.

## Figures and Tables

**Table 1:** General characteristics of the population.

	Total Population n = 90
Age years m (SD)	31.2 (8.86)
Female sex n (%)	54 (60)
Educational level n (%)	
Student	30 (33)
Professional	60(67)
Health student, n (%)	
Medicine	18 (60)
Postgraduate medicine or nursing	12 (40)
Health professional, n (%)	
Medicine	20 (33)
Nursing	27 (45)
Other	13 (22)
CPR training, n (%)	68 (76)
CPR experience, n (%)	52 (58)

Notes: n: number; CPR: cardiopulmonary resuscitation; m: media; SD: Standard deviation.

**Table 2:** Perception of performing high-quality compressions.

	Without Guardian Angel n = 90	With Guardian Angel n = 90	*p* Value
Perception of performing high-quality compressions, n (%)	57 (63)	87 (97)	<0.001
High-quality compressions in frequency (100 to 120) in infants, n (%)	17 (19)	77 (85)	<0.001
High-quality compressions in frequency (100 to 120) in adults/schoolchildren, n (%)	23 (25)	71 (79)	<0.001

Notes: n: number; CPR: cardiopulmonary resuscitation.

**Table 3:** Perception of performing high-quality compressions in participants with training, experience and fatigue.

	Without Guardian Ángel n = 90	With Guardian Ángel n = 90	*p* Value
CPR training n = 68 (76)			
High-quality compressions in depth (≥70) in the infant	4 (6)	61 (90)	<0.001
High-quality compressions in depth (≥70) in schoolchildren/adults	18 (26)	65 (96)	<0.001
High-quality compressions in frequency (100 to 120) in the infant	17 (25)	64 (94)	<0.001
High-quality compressions in frequency (100 to 120) in schoolchildren/adults	23 (34)	62 (91)	<0.001
No CPR training n = 22 (24)			
High-quality compressions in depth (≥70) in the infant	0 (0)	8 (36)	0.008
High-quality compressions in depth (≥70) in schoolchildren/adults	0 (0)	10 (45)	0.002
High-quality compressions in frequency (100 to 120) in the infant	0 (0)	13 (59)	<0.001
High-quality compressions in frequency (100 to 120) in schoolchildren/adults	0 (0)	9 (41)	0.004
CPR experience n = 52 (58)			
High-quality compressions in depth (≥70) in the infant	48 (8)	48 (92)	<0.001
High-quality compressions in depth (≥70) in schoolchildren/adults	15 (29)	50 (96)	<0.001
High-quality compressions in frequency (100 to 120) in the infant	15 (29)	50 (96)	<0.001
High-quality compressions in frequency (100 to 120) in schoolchildren/adults	20 (38)	50 (96)	<0.001
No experience in CPR n = 38 (42)			
High-quality compressions in depth (≥70) in the infant	0 (0)	21 (55)	<0.001
High-quality compressions in depth (≥70) in schoolchildren/adults	3 (8)	25 (66)	<0.001
High-quality compressions in frequency (100 to 120) in the infant	0 (0)	13 (59)	<0.001
High-quality compressions in frequency (100 to 120) in schoolchildren/adults	0 (0)	9 (41)	<0.001
Start compressions with CPR device n = 36 (40)			
High-quality compressions in depth (≥70) in the infant	3 (8)	28 (78)	<0.001
High-quality compressions in depth (≥70) in schoolchildren/adults	10 (28)	31 (86)	<0.001
High-quality compressions in frequency (100 to 120) in the infant	8 (22)	32 (89)	<0.001
High-quality compressions in frequency (100 to 120) in schoolchildren/adults	12 (33)	33 (92)	<0.001
Start compressions without CPR device n = 54 (60)			
High-quality compressions in depth (≥70) in the infant	1 (2)	41 (76)	<0.001
High-quality compressions in depth (≥70) in schoolchildren/adults	8 (15)	44 (81)	<0.001
High-quality compressions in frequency (100 to 120) in the infant	9 (17)	45 (83)	<0.001
High-quality compressions in frequency (100 to 120) in schoolchildren/adults	11 (20)	38 (70)	<0.001
Without reference to fatigue or mild fatigue n = 68 (75)			
High-quality compressions in depth (≥70) in the infant	3 (4)	55 (81)	<0.001
High-quality compressions in depth (≥70) in schoolchildren/adults	15 (22)	59 (87)	<0.001
High-quality compressions in frequency (100 to 120) in the infant	14 (21)	61 (90)	<0.001
High-quality compressions in frequency (100 to 120) in schoolchildren/adults	18 (26)	56 (82)	<0.001
With reference to moderate or severe fatigue n = 22 (24)			
High-quality compressions in depth (≥70) in the infant	1 (4)	14 (64)	<0.001
High-quality compressions in depth (≥70) in schoolchildren/adults	3 (14)	16 (73)	<0.001
High-quality compressions in frequency (100 to 120) in the infant	3 (14)	16 (73)	<0.001
High-quality compressions in frequency (100 to 120) in schoolchildren/adults	5 (23)	15 (68)	0.02

Notes: n: number; CPR: cardiopulmonary resuscitation.

## Data Availability

The original contributions presented in this study are included in the article/Supplementary Material. Further inquiries can be directed to the corresponding author.

## Supplementary Materials

The following supporting information can be downloaded at https://www.mdpi.com/article/10.3390/jcm15082839/s1; Figure S1: The general characteristics of the Sharp^®^ GP2Y0A21YK.
