# Supplementary material for: Clinical Utility of a Feedback Device in High-Quality Cardiopulmonary Resuscitation: The Guardian Angel
Source: J Clin Med. 2026 Apr 9;15(8):2839. doi: 10.3390/jcm15082839 (PMC13115935; doi:10.3390/jcm15082839)
Supplement: Supplementary file 1 [file jcm-15-02839-s001.zip › jcm-4179688-supplementary.pdf]

Figure S1. The general characteristics of the Sharp® GP2Y0A21YK.

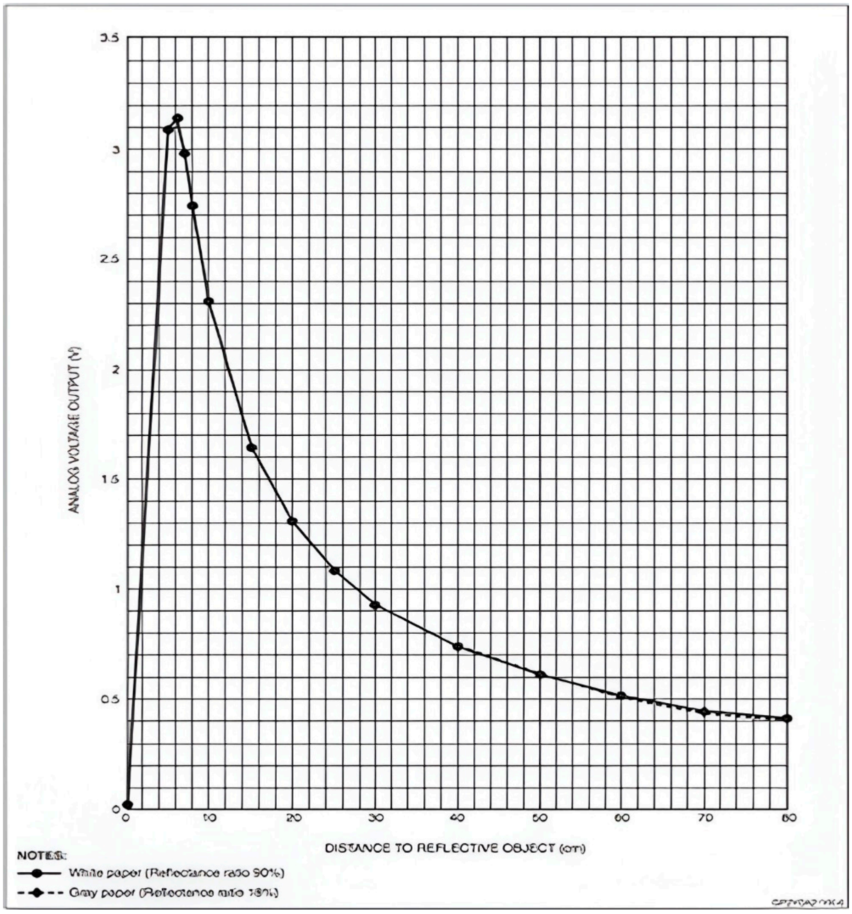

Notes: The Sharp® GP2Y0A21YK is an optical distance sensor, composed of an infrared light-emitting diode (LED) emitter that emits a pulsed infrared light beam (wavelength of 850 nm +/- 70 nm) along with a position detecting device. We employ the principle of triangulation as a measurement method to determine the distance from the sensor to objects located in front of the beam. It consists of a 3-pin Japanese Solderless Terminal connector: power, ground, and output. The latter provides an analog value (voltage) according to the distance of the detected object, ranging between 0.4 volts (at 80 cm) and 3.1 volts (at 10 cm) depending on the measured distance, as per the curve provided by the manufacturer in its datasheet.
